# Functional Brain Networks: Random, “Small World” or Deterministic?

**DOI:** 10.1371/journal.pone.0078763

**Published:** 2013-10-30

**Authors:** Katarzyna J. Blinowska, Maciej Kaminski

**Affiliations:** Department of Biomedical Physics, Faculty of Physics, Warsaw University, Warsaw, Poland; University of Michigan, United States of America

## Abstract

Lately the problem of connectivity in brain networks is being approached frequently by graph theoretical analysis. In several publications based on bivariate estimators of relations between EEG channels authors reported random or “small world” structure of networks. The results of these works often have no relation to other evidence based on imaging, inverse solutions methods, physiological and anatomical data. Herein we try to find reasons for this discrepancy. We point out that EEG signals are very much interdependent, thus bivariate measures applied to them may produce many spurious connections. In fact, they may outnumber the true connections. Giving all connections equal weights, as it is usual in the framework of graph theoretical analysis, further enhances these spurious links. In effect, close to random and disorganized patterns of connections emerge. On the other hand, multivariate connectivity estimators, which are free of the artificial links, show specific, well determined patterns, which are in a very good agreement with other evidence. The modular structure of brain networks may be identified by multivariate estimators based on Granger causality and formalism of assortative mixing. In this way, the strength of coupling may be evaluated quantitatively. During working memory task, by means of multivariate Directed Transfer Function, it was demonstrated that the modules characterized by strong internal bonds exchange the information by weaker connections.

## Introduction

It is more and more acknowledged that progress in understanding of information processing in the brain depends to the large degree on evaluation of temporal and spatial patterns of connections between neural populations. The evidence has accumulated that coupling between neural populations serves brain function integration involving the transient synchronization between distant and specific neural populations e.g.: [1], [2].

The problem of the determination of the connectivity structure in the brain has been a subject of intense research in the last years. The methods used for estimation of connectivity include bivariate methods such as: correlation, coherence, Mutual Information (MUI), Synchronization Likelihood (SL), Transfer Entropy or multivariate methods such as Directed Transfer Function [3] and Partial Directed Coherence (PDC) [4]. The above mentioned multivariate methods based on Granger causality principle supply the spectral information and the information about directionality of interaction (so called effective connectivity). Among bivariate methods: correlation (directionality can be found from the time delay of cross-correlation function), coherence (directionality can be found from the phase of coherence) and Transfer Entropy have a potential to indicate the directionality, but in many cases this information is neglected. Usually connectivity patterns obtained by bivariate methods are characterized by a very dense structure, without the distinct topographical features and hence they require further analysis.

In the last years, for the analysis of connectivity based on EEG data, graph theoretical analysis has been widely applied. The formalism introduced by Watts and Strogatz [5] and developed further in e.g. [6] or [7] involved determination of the measures characterizing networks. The most commonly used among them are: node degree, clustering coefficient and characteristic path lengths of networks. These parameters served for distinguishing regular or random network from the “small world” organization, which is characterized by a high clustering coefficient and a short path length.

Multiple EEG and also MEG studies, involving graph theoretical approach, for different experimental paradigms pointed out to the presence of “small world” structures in the brain e.g.: [8], [9], [10], [11], [12], however quite often the obtained pattern of connections hardly differed from randomness.

It seems that the time is ripe to ask: 1) what we can learn from graph theoretical analysis based on bivariate measures, beside the degree to which network is “small world” or random and 2) how well the results going beyond random or ”small word” dilema obtained by this methodology correspond to the evidence obtained from other sources: e.g.: coming from anatomical, imaging, and inverse solution studies.

Inspecting the works based on bivariate measures and graph theoretical analysis, it is difficult to find the correspondence to the imaging and electrophysiological evidence. As an example may serve the finger movement experiment [10], where no statistically significant changes in network parameters depending on frequency were found between resting and motor state and no lateralisation was observed (lateralisation in this context means that the biggest changes in brain activity occur at the side contralateral to the hand/foot performing the movement). Another example of the lack of correspondence between networks configuration obtained by bivariate measures of connectivity (followed by the graph theoretical analysis) and other evidence, stems from studies involving sleep EEG, where no significant changes in network parameters were found for different sleep stages [8], [11]. Contrary to these findings the clear-cut changes in connectivity during sleep were demonstrated in [13]. Similarly, in working memory (WM) task studied by means of synchronization likelihood and graph theoretical analysis [12] no correspondence to known imaging [14], [15] and electrophysiological [16] evidence was found. It seems surprising that the localization of the cortical sources and known interactions between them are not reflected in connectivity patterns studied by theoretical graph analysis based on bivariate measures of connectivity. Although the anatomical connections form a very dense matrix, the physiological evidence indicates that in specific tasks only some very specific connections are active. In our opinion the reasons of these discrepancies merits the attention.

Recently there appeared publications raising important questions concerning methodology standing behind the graph theoretical analysis application to EEG and MEG signals. They involved in particular the influence of common source ([17], [18], [19]), the problem of volume conduction ([17], [18], [20]), choice of threshold ([20]) and the effect of network size [21].

Herein we shall try to approach some of these problems. In our opinion the crucial topic here is the choice of the connectivity estimator, which takes into account the influence of the common source. In most works applying graph theoretical analysis connectivity was estimated pair-wise. The difference between multichannel and pair-wise approach to the problem of estimation of connectivity patterns was considered in: [22], [23]. The results pointed out unequivocally that the pair-wise estimation of connectivity produces spurious connections. In the following we shall compare connectivity patterns obtained by means of bi-variate measures with these obtained by means of multivariate estimator by means of: simulations, analysis of experimental signals and examples from the literature.

## Methods

We shall only briefly describe the methods of connectivity estimation used in this paper, since they can be found in the literature e.g.: [24], [25], [26].

Lately, for estimation of connectivity quite often synchronization likelihood [27] has been used. Synchronization likelihood (SL) relies on construction of vectors from given time series by time-delay embedding. The SL describes a chance that pattern recurrence present in the embedded vector constructed from time series *X* coincides with the pattern recurrence in the embedded vector of signal *Y*. The concept of synchronization likelihood is closely related to the definition of Mutual Information (MUI)—measure based upon the correlation integral [28]. The difference is, that SL is normalized.

To estimate the connectivity in the framework of multivariate formalism, we shall use the Directed Transfer Function [3]. The Directed Transfer Tunction (DTF) is a measure based on the Granger causality concept. It is defined in the framework of multivariate autoregressive model (MVAR). The model is simultaneously fitted to all channels of the set. DTF describes causal influence of channel *j* on channel *i* at frequency *f* according to the formula:
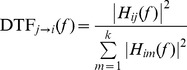
(1)


where *H_ij_*(*f*) is an element of the transfer matrix of the MVAR model. The above equation defines a normalized version of DTF, which takes values from 0 to 1 producing a ratio between the inflow from channel *j* to channel *i* to all the inflows to channel *i*.

Time varying connectivity may be described by the Short-time Directed Transfer Function (SDTF). It is a modification of DTF, which may be applied when multiple repetitions of experiment are available. Then the combination of ensemble averaging with a short sliding window makes possible to estimate dynamic propagation [26], [29], [30].

For the identification of the community structures of the network the method of assortative mixing was introduced [31]. In the framework of assortative mixing approach the element *E* ***_kl_*** of the connectivity matrix **E** is defined to be a fraction of edges in a network that connects a vertex of group *k* to one of group *l*. In our case indexes *k* and *l* do not refer to the channels present in the definition of DTF, but to the groups defined in the framework of assortative mixing. (These groups encompass integrated DTFs as will be explained below). In an undirected network matrix **E** is symmetric in its indices *E_lk_* = *E_kl_*, while in directed networks it may be asymmetric. Mixing is highly assortative when the diagonal elements of matrix **E** are significantly higher than the off-diagonal ones. It corresponds to the situation of strongly connected modules, with weaker bonds between these modules.

In our case the elements of matrix **E** are represented by the propagations (DTFs) either inside the module (*E_kk_*), or between the modules (*E_kl_*). The **E** matrix is calculated according to the formula:
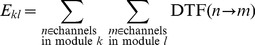
(2)


where DTF(*n*→*m*) denotes average value of DTF*_n_*_→*m*_(*f*) in the given frequency range. For SL, matrix **E** calculated in a similar way is symmetric. The resulting matrices were normalized in such a way that the sum of all its elements is equal to 1.

Considering the elements of matrix **E** we may deduce, if our case corresponds to the situation of highly assortative mixing *E_kk_>E_kl_*. If indeed it is so, we can say that the connectivity is stronger inside the modules than between the modules.

## Results

Let us first consider, by means of a simulation, a common situation that the source emits its activity, which is registered at several electrodes located at different distances. The simulation scheme was designed in the following way: the signal in channel 1 was constructed from an experimental EEG (2560 samples long, sampled at 128 Hz, highpass filtered with cutoff frequency 3 Hz) plus a random noise. The signals in destination channels were constructed by introducing delays and adding to each delayed channel an extra noise (drawn from different random noise generators). The connectivity patterns were estimated by means of bi-variate coherences and by DTF.

**Figure 1:**
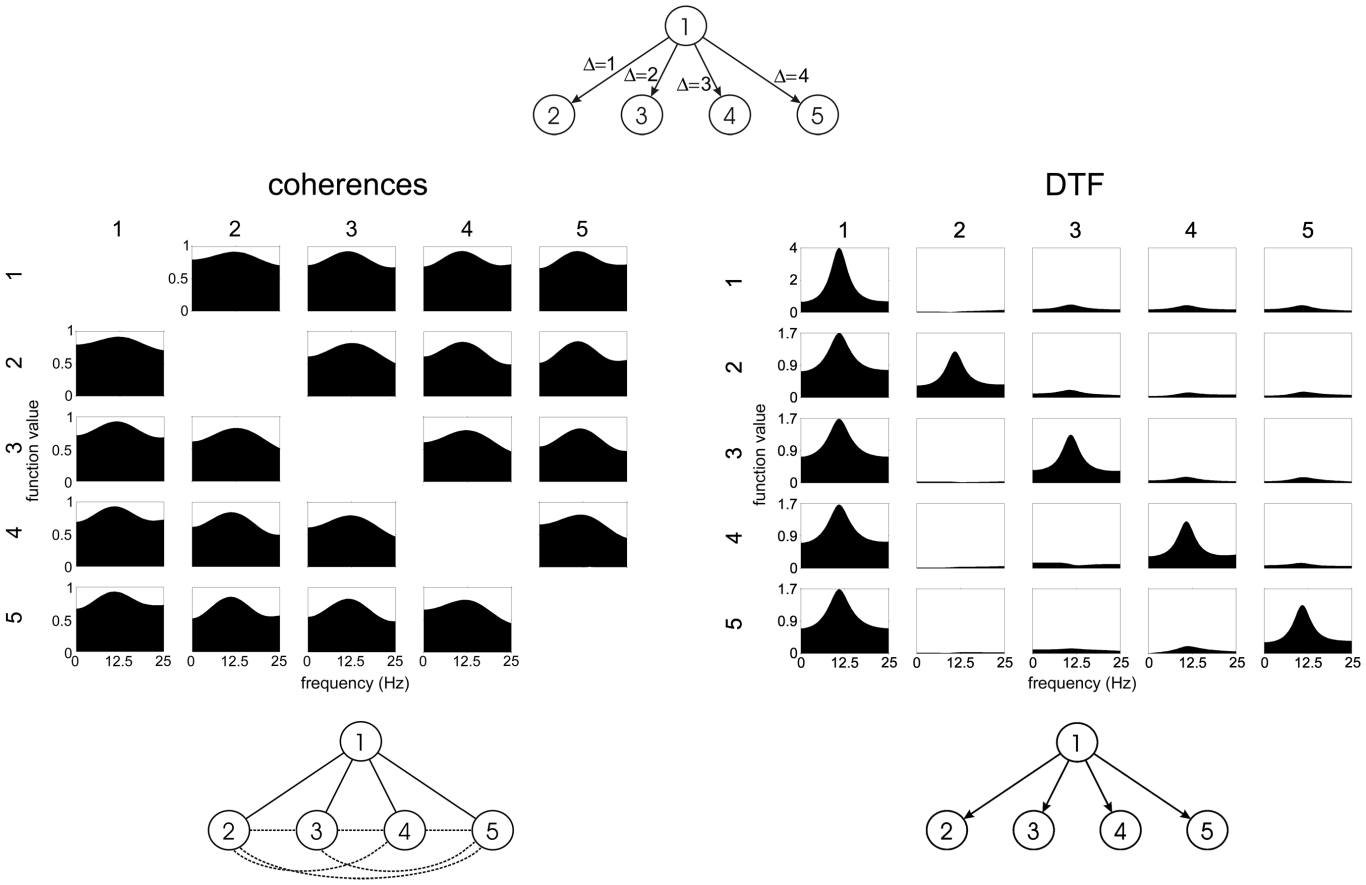
Simulation description and results. Top: simulation scheme, signal from channel 1 is propagating to the other channels with different delays. Graphs—left: ordinary coherences; right: multivariate DTFs as functions of frequency. Propagation by DTF is shown from the channel marked above to the channels marked at the left of the picture (on the diagonal power spectra). At the bottom obtained corresponding connectivity schemes. Incorrect flows shown by broken lines.

The results of such simulation are illustrated in Fig. 1. It is easy to see, that not only existing connections will be found by a bi-variate estimator, but also a lot of spurious connections emerge. In the simulation coherences were used, however the results will be practically the same for every bivariate estimator. The simulation was performed for directed connectivity, but results hold as well for undirected connectivity, since the same spurious connections as the ones illustrated in Fig. 1, will be found for undirected estimator, in virtue of common feeding to different channels.

In fact, it is easy to estimate the ratio of true to false connections. Namely, when we register activity from a given source at *N* electrodes, *N* true and *N*(*N*–1)/2 false connections may be found by a pair-wise estimate. We should note that in the case of recording activity by *N* electrodes from a given source number of false connections increases as *N* ^2^, whereas a number of true connections increases only as *N*. When two or more sources are simultaneously active, very dense and disorganized pattern of connections may be found by pair-wise estimators, as reported in many papers, where such estimators were used.

**Figure 2:**
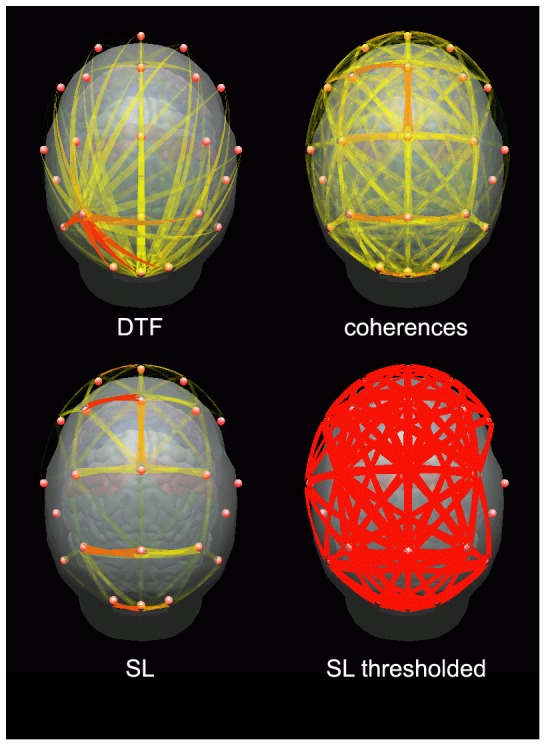
Connectivity patterns obtained for awake state, eyes closed by: DTF (upper left), bivariate coherences (upper right), SL (lower right), pattern obtained from SL by giving all connections equal weight (threshold = 0.2).

Herein we shall compare connectivity patterns obtained from EEG data by means of coherences, SL and DTF for resting state EEG and for working memory task (data are available at http://brain.fuw.edu.pl/~kjbli/Data_pack_PLOSONE2013a.zip). In Fig. 2 the connectivity patterns for frequency range 0.5 Hz to 40 Hz obtained by: DTF (MVAR fitted simultaneously to all channels, model order 5), coherences and SL (delay time: 10 samples, embedding dimension: 10 samples) are shown for the resting state EEG, eyes closed. In case of DTF the propagation from the posterior electrodes is prominent. This kind of pattern might be expected in the light of previous studies on the group of subjects [13] and from the known fact that in the resting state, eyes closed the EEG activity is mostly generated in visual cortex [32].

In case of ordinary coherences the pattern is very dense and almost uniform. For SL there are more connections in the frontal than in the posterior region, which can be explained by the fact that common feeding from posterior to frontal electrodes generates multitude of false connections between frontal electrodes (compare Fig. 1). When all connections found by means of SL (of intensity higher than 0.2) were given equal weights a very dense pattern of connections emerges (bottom left corner of Fig. 2). We have applied threshold 0.2, to construct this plot, since for the lower threshold the network would be so dense, that it would be impossible to distinguish particular connections in the picture. However, usually the thresholds applied in graph theoretical analysis are lower — between 0.01 to 0.05 [9], [12]. Sometimes the thresholds are adapted in such a way, that a resulting network is fully connected and the number of connections per vertex is the same for all subjects and conditions e.g.: [8], [11].

**Figure 3:**
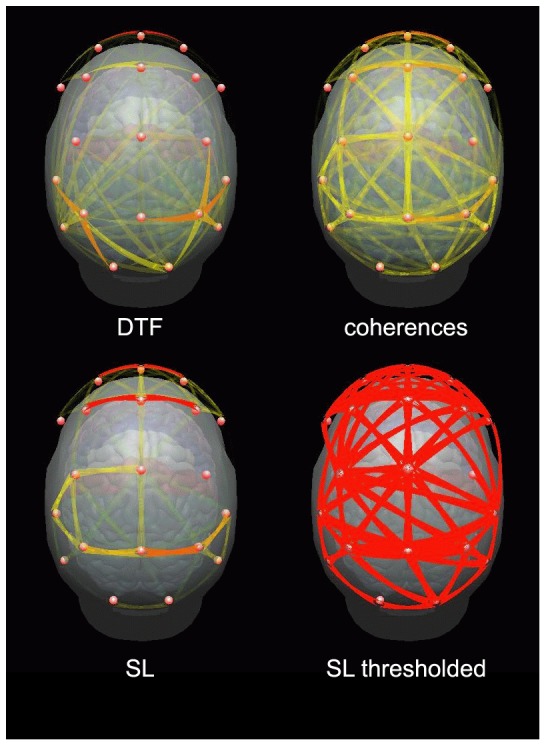
Connectivity patterns obtained for a working memory task by DTF (upper left), bivariate coherences (upper right), SL (lower right), pattern obtained from SL by giving all connections equal weight (threshold = 0.2).

In Fig. 3 connectivity patterns obtained by means of DTF, coherences and SL are shown for a working memory task for one subject. The task involved memorizing and retrieval of the sequences of letters; the description of the paradigm may be found in [33]. Connectivity values were integrated in the 1–45 Hz frequency range and in the epoch 0–3 s after presentation of the stimulus. In case of DTF we can observe short-range propagation from posterior and frontal sources. (In time-varying connectivity patterns obtained by SDTF also long-range connections are visible, but they appear only in certain moments [34], so they are not visible in the picture integrated over whole epoch).

In case of coherences the connectivity structure is dense and similar over the whole scalp. For SL the pattern bears some similarity to DTF, but connections are present also in the central region and they are stronger in frontal locations than in the posterior ones. This pattern may be explained (as in the case presented in Fig. 2) by connections produced by common feeding. When the connections are given equal weights as is customary in graph theoretical analysis, a very dense connectivity pattern emerges.

One of the measures used in graph theoretical analysis is the node degree. In case of directed connectivity the numbers of outgoing and ingoing connections to the node may be estimated. In order to define the threshold for significant connections obtained by DTF we have applied bootstrap method [35]. The threshold value differed to certain degree between channels, the highest value which corresponded to 0.9 significance level, was 0.0454. We have fixed the threshold at the value 0.05 for all channels and the same cutoff value was used for the SL data, since the usual thresholds applied in SL studies are around this value.

**Figure 4:**
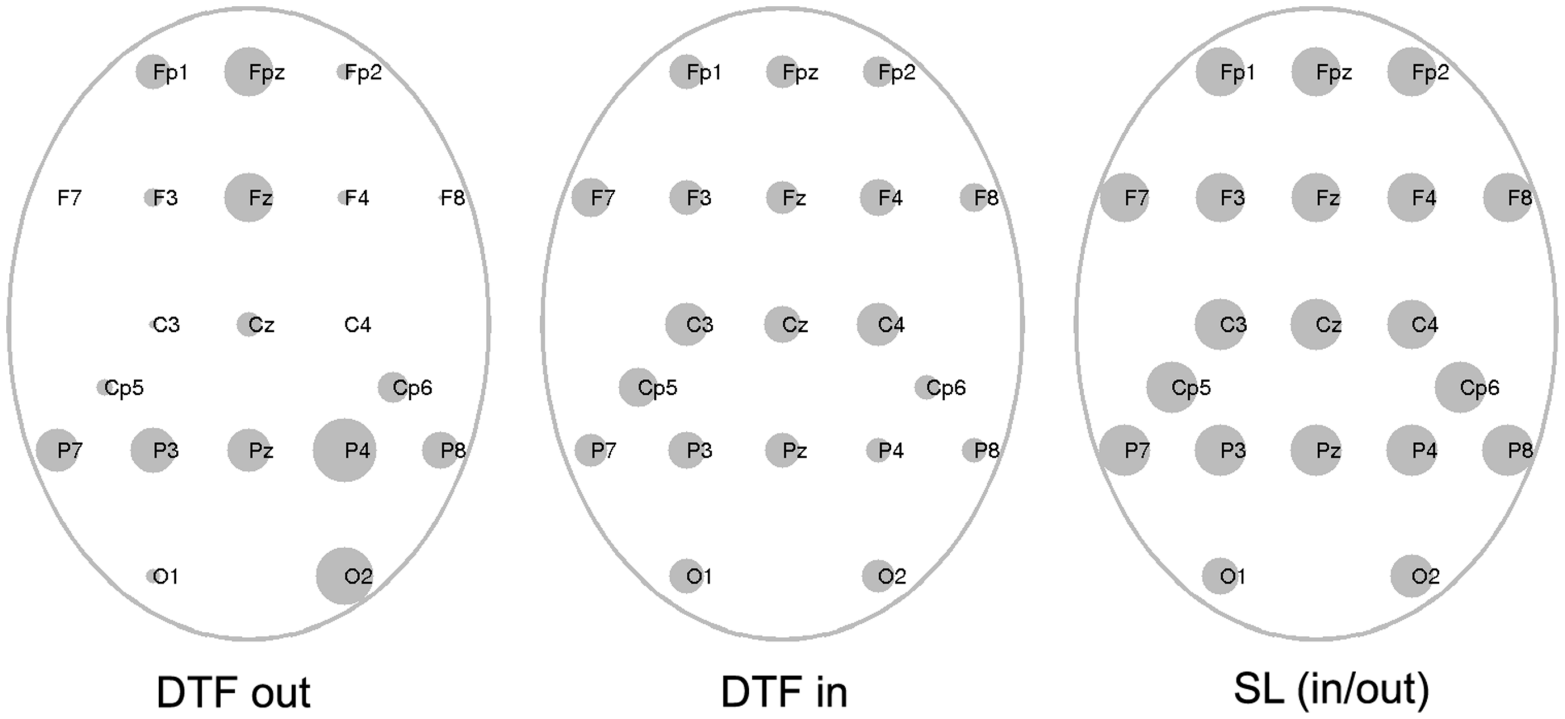
Node degrees for DTF and SL coded by the dimensions of the circles. From left to the right: DTF outflows, DTF inflows, SL node degree.

The results are shown in Fig. 4. For the DTF outflows we can see that the strongest nodes are located in the right parietal and occipital locations, also in front, which is in agreement with the position of sources found by means of imaging studies [14], [15]. The pattern of the inflows is mostly uniform and does not indicate existence of the distinct sinks. For SL there are very small difference between the strengths of the nodes, which may be explained by the multitude of the connections (true and false) produced by the bivariate measures.

For very sparse networks as the ones obtained by means of DTF the classical measures such as clustering coefficient and path length are not appropriate. However we may use more advanced measures of modularity such as assortative mixing, which allows for distinguishing the modules within which connections are dense, but between them they are sparse. Inspecting Fig. 3 and Fig 4 we may distinguished four main modules: frontal F involving electrodes Fp1, Fp2, Fpz, F3, F4, Fz, F7, F8; central C: (C3, Cz, C4, Cp5, Cp6) and two parietal—PL: (P7, P3, Pz, O1) and PR: (P8, P4, Pz, O2).

The arguments for the choice of the above defined modules were based on: obtained patterns of transmissions, node degrees, the imaging [14], [15] and physiological evidence [36], [37], [38] concerning the role of frontal and posterior structures.

In order to construct the matrix **E** we integrated DTFs in 4–60 Hz frequency band and in the time epoch 0–3 s, and we calculated the matrix **E** according to the eq. 2. The elements *E_kk_* corresponded to the connection strength inside the module, that is, DTF values between close electrodes, belonging to the same group e.g.: for parietal left cortex—connections between electrodes: P7, P3, Pz, O1. Elements *E_kl_* corresponded to the DTFs between electrodes belonging to different modules. The same procedure was applied in case of SL, the only difference was that in this case elements *E_kl_ = E_lk_*. For both estimators the connections above the threshold 0.05 were taken into account.

**Figure 5:**
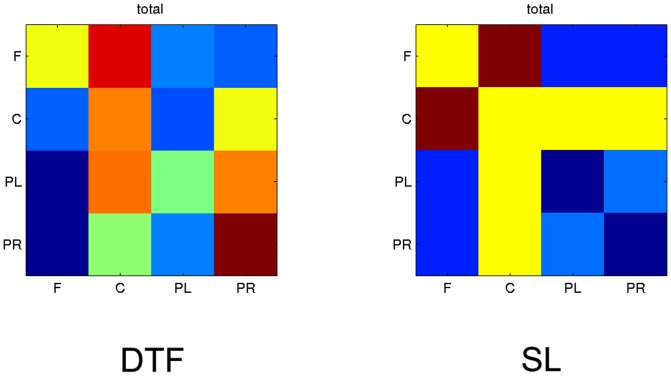
The results of assortative mixing for four modules in the whole (4–60 Hz) frequency band. At the left for DTF, at the right for SL. On the diagonal coupling within the modules, off-diagonal between the modules. For DTF the causal coupling from the module marked below the column of boxes to the module marked at the left. In each box the strength of coupling is illustrated in color scale: dark red-the strongest, dark blue-the weakest.

The results (integrated in whole frequency band) showing the percentage of coupling strength (errors are included), are shown in Table 1 and in Fig. 5 as a matrices of color boxes. On the diagonal of the matrices the strength of coupling inside the module and off-diagonal strength of coupling between the modules are illustrated. It is easy to see that in the case of DTF, the coupling within the modules is mostly stronger than between them. For SL strong coupling between central and other regions may be observed, especially between central and frontal module. Coupling within and between parietal regions is weak. Strong coupling within central module and between central and other modules may be explained by the fact that the propagation from frontal and posterior sources was directed toward that region which produced a lot of spurious connections between central electrodes. The results shown in Fig. 5 were obtained for one subject. In [34] the assortative mixing was applied for DTF functions of 12 subjects and the averaged results pointed out unequivocally that the coupling within the modules is stronger than between different modules.

**Table 1:** Assortative mixing values for four modules presented in Fig. 5.

DTF					SL				
	F	C	PL	PR		F	C	PL	PR
F	8%	12%	3%	3%	F	7%	9%	5%	5%
C	3%	10%	3%	8%	C	9%	7%	7%	7%
PL	0%	10%	7%	10%	PL	5%	7%	4%	5%
PR	0%	7%	3%	13%	PR	5%	7%	5%	4%

### Comparison of connectivity studies conducted by means of pair-wise methods followed by graph theoretical analysis with multivariate studies

The illustrative problem, which has been studied by means of different approaches, is a study of a finger movement task. It was also approached by means of the graph theoretical analysis. The self paced finger movements recorded by means of magnetoencephalographic (MEG) technique was analyzed by means of wavelet analysis followed by calculation of correlations and graph theoretical analysis (the network characteristics was considered in the range of thresholds: 0.4≤τ≤0.8) [10]. Connectivity in different frequency bands was evaluated by means of correlation between pairs of sensors. “Small world” topography was found, however the specific parameter values such as clustering coefficient and characteristic path length did not show statistically significant differences between frequency bands and resting/motor state. In the network topology (spatial distribution of highly connected network nodes) no lateralisation was observed, although it is a well known phenomenon, confirmed in all studies dealing with motor tasks. As the main difference between resting and motor activity authors reported emergence of a new long range connections and pivotal nodes in frontal and parietal regions in the gamma and beta networks, although the global topological parameters of these networks were not much affected by the change of the behavioral state.

It is difficult to find the correspondence of this study with the results obtained in other works concerning finger movement task. The topographical and spectral features of the movement tasks were studied thoroughly by Pfurtscheller [39], who found the desynchronisation (decrease of power in alpha and beta band during movement in the primary motor (MI) area contralateral to the moving finger) and short burst of gamma activity in this location. In the simultaneous fMRI/EEG study [40] contralateral primary motor (MI) and primary sensory cortex activation was reported from fMRI experiment. The inverse solution method applied in the above study yielded the dipole localization in MI consistent with fMRI results.

In the presence of the sources identified in the above studies one may expect that the activity propagation from them should strongly influence the topography of networks, mainly in the central regions of the cortex, where the motor areas are located, also the lateralisation of the brain activity (well known from anatomical and experimental evidence) should have an impact on connectivity. Nothing like that was observed in [10].

The EEG studies based on multivariate autoregressive model (MVAR model orders 5–7), namely the effective connectivity patterns obtained by means of Short-time Directed Transfer Function [29], [41], [42] showed an excellent agreement with the imaging and inverse solution studies. The phenomenon of desynchronization in alpha and beta band [39] was visible as the gap in the propagation in these bands from electrodes overlying contralateral MI in respect to the moving finger. The burst of gamma propagation from contralateral MI was accompanying movement and in case of movement imagination the cross talk between MI and other sensorimotor structures was observed (animations available at http://brain.fuw.edu.pl/~kjbli/DTF_MOV.html). These results are in contradiction to findings of Basset et al. [10] that dynamic properties of brain functional networks during motor task are conserved over frequencies.

Synchronization likelihood, followed by graph theoretical analysis, was applied for sleep studies by Leisted et al. [11]. The number of connections per vertex of a graph was arbitrarily set to 5. The authors compared obtained network parameters with the random graphs. The reported characteristic path lengths did not differ from random graphs, clustering coefficients did differ, but no dependence of their values on the frequency band was found. The pattern of connections in the delta band was very dense and disorganised and did not show any *hubs* (hub—node of the network connected to many other nodes).

Another example of the discrepancy of works based on graph theoretical analysis with other evidence may be the work of Ferri et al. [8] who similarly to [11] for the study of connectivity during sleep and wakefulness used SL and graph theoretical analysis. No significant differences were found between sleep stages. The only difference between sleep and wakefulness was higher clustering coefficient in sleep.

The above results are in the contradiction to the study of the effective connectivity for wakefulness and sleep performed by means of DTF for a group of 9 subjects [13]. In this study dramatic differences in connectivity between sleep stages were found. In wakefulness sources of EEG activity were predominant in parietal or occipital areas (as in the case illustrated in Fig. 2). In stage 1 there was an increase of propagation in the frontal area, in stage 2 two sources of activity - frontal and parietal were active and in the deep sleep the prominent source located over *corpus callosum* was sending the propagating activity over a whole brain (see: http://eeg.pl/DTF/dtf-mapping-versus-dtf). These results were very repeatable for all studied subjects. They are compatible with the physiological knowledge concerning sleep processes. The pattern of connectivity for deep sleep shown in ref. [11] seemed to be random, it did not show any *hubs*, any highly connected network nodes.

The brain networks in wakefulness, eyes closed and open were investigated in [43]. The local measures called centrality indexes were considered. *Strengths centrality* was defined as a number of links incident upon a node. This index showed increased importance of frontal brain sites and a dcreased importance of parieto-occipital brain sites during eyes closed state, which is rather surprising result, taking into account preponderance of the parieto-occipital region as strong source of activity during eyes closed state.

The EEG in eyes closed condition was also investigated in the work [44]. The authors recognized the influence of common source influence and they applied not only odinary (unpartial) but also partial correlation in their studies. From their analysis conducted on simulated and EEG data it followed that nework properties are quite different for two kinds of correlations. In particular number of over-identified links was much higher for unpartial correlations. However the estimation of partial correlaions is cumbersome and computationaly expensive as the authors admitted themselves. On the other hand elimination of the influence of common source by calculation of estimators such as partial coherences or DTF in the framework of MVAR is straightforward.

Working memory task, so called 2Back check, relying on memorizing and recollection of Greek letters was applied in [12] to the group of less and better educated people. EEG data were analyzed by means of SL followed by graph theoretical analysis. The authors considered the range of threshold values 0.01 – 0.05, which corresponded to the number of edges 4–5. Between-group differences based on the clustering coefficient and path length were observed, but did not reach statistical significance. For less educated people “small world” connectivity was found, for better educated group the connectivity pattern was close to random in all frequency bands No topographic differences or differences concerning changes of connectivity in specific frequency bands between resting state and working memory task were reported.

Two-Back check paradigm was also applied in the work of Kitzbichler et al. [45] who estimated synchronization by means of phase differences between MEG sensors. The threshold values were based on predetermined connection density, which was initialy fixed at 10%, but was also considered in the range of values: between 2% and 20%. Local and global efficiency, node degree and the geometrical network distance were estimated. The main findings concerned increase of the long-range connections for the more difficult (2-back tasks). For these tasks the decrease of synchronization and close to random structure of the networks was observed. Task dependent differences in networks organization quantified by node degree, global and local efficiency and the physical distance indicated more random network architecture for more difficult task. The pattern of connections was very dense and did not indicate the brain regions involved in the information processing during working memory tasks. The stronger node degrees (reported in the beta band) for more difficult task were located mainly along the midline of brain.

This results may be compared with connectivity study [33] concerning working memory task, which relied on memorization of the letters and relations between them and then retrieval of this relation. The retrieval of relations was considered to be difficult task in comparison to the memorization. The connectivity was calculated by means of DTF function and the significant differences between easy and difficult tasks were determined according to procedure proposed in [46]. For more difficult task the increase of long range connections were found, but contrary to [45] these long range connections were well specified and the patterns of connectivity were far from randomness. Namely in the theta and alpha bands they were directed from the frontal toward posterior regions. In the gamma band the propagation from right parietal to frontal region was observed. Also increase of short-range connectivity was found in right parietal region.

The dynamic changes of connectivity during working memory task were studied by means of SDTF in [34]. In this study the patterns of dynamical connectivity were established; the animations showing the time-varying pattern of connectivity are available at: http://brain.fuw.edu.pl/~kjbli/Cognitive_MOV.html. The results of DTF and SDTF studies, in excellent agreement with the evidence from imaging and electrophysiological experiments, showed fronto-parietal regions involvement in working memory processes. Moreover the SDTF study revealed strong coupling within frontal and parietal regions. The coupling between these two centers occurred only during certain short epochs. The ratios of the strength of short-range to long-range connections were of the order of 1.5 [34].

The above described connectivity patterns obtained for working memory tasks may be confronted with the other evidence. The neuroimaging experiments showed the involvement of frontal and parietal regions in working memory task [14], [15]. Right prefrontal and bilateral parietal cortex were shown to be active in tasks with nonspecific stimuli (lacking conceptual content, like letters) [37], [38]. Moreover, an increase in the magnitude of frontal theta oscillations and increased frontal-posterior theta coherence reflect the executive functions of working memory system [16]. Kawasaki and Watanabe [47] showed that the gamma-band increase in the frontal and parietal regions is associated with manipulation of visual mental representations, particularly successive ones. The above quoted evidence is in excellent agreement with the connectivity patterns found by means of DTF and SDTF. Results obtained in [33] and [34] corroborate other evidence and enrich the knowledge concerning working memory processes by supplying the information on directionality of transmissions, ratio of short to long connections and the dynamics of the processes.

Good topographical agreement of connectivity patterns obtained by DTF is connected not only with the absence of spurious connections, but also with the fact that DTF is practically not influenced by the volume conduction. DTF (as well as PDC) is based on phase difference betweensignals of the multivariate set. Volume conduction as a propagation of electromagnetic field doesn’t generate phase difference on the electrodes. The fact that DTF is not influenced by the zero phase propagation was demonstrated by means of simulation (http://en.wikipedia.org/wiki/Brain_connectivity_estimators). The fact that methods based on phase differences are immune to volume conduction was recognized e.g. in [48]. We can see that the second important problem – influence of volume conduction - raised in methodological publications concerning the difficulties encountered in graph theoretical analysis may be solved by application of proper multivariate method.

## Discussion

The above results show that by giving the equal weights to all connections between electrodes we lose some information, but we gain the possibility to use graph theoretical analysis in its classical form introduced in [5]. What do we learn from this analysis? We learn that in most cases there exists some “small word” structure in connectivity. What else do we learn? By the inspection of the works where graph theoretical analysis was used, we can say that the formalism gives us usually very general information, namely, it does not give the information where the centers of activity, presumabely indicated by a “small word” structure, are localized. The localization of nodes of high degree or high local efficiency usually does not give information consistent enough to indicate the centers engaged in a given task. The attempts to find the frequency dependence of the connectivity usually give results, which have no correspondence with the other electrophysiological information.

In multiple papers applying graph theoretical analysis the main finding was a presence of “small world” connectivity in brain networks. Can we identify “small world” structure by means of multivariate measures? Unfortunately, the notion of “small world” structure is ill defined for networks identified by multivariate methods, since in the formalism of graph theoretical analysis it is assumed, that every vertex should be connected to the other one at least by one path and small number of steps. This assumption does not hold for multivariate effective connectivity, since the networks are very sparse. It is possible to relax the assumption of full connectivity by introduction of global efficiency, which is an inverse of the shortest path length [7]. Then for disconnected nodes one assumes zero efficiency. However the networks obtained by multivariate methods are usually so sparse that overwhelming part of the connections will take zero value and the global efficiency and clustering coefficient will be always very low.

However, for quantification of networks we can adopt more advanced analysis based on assortative mixing. This approach takes into account the directedness and strengths of connections and allows for finding coupling inside and among the modules. The application of this method to the connectivity determined by means of DTF indicated, that for the working memory task the strength of connections inside the modules tends to be stronger than between modules.

The above results show that even when we abandon the exact definition of “small world” networks we can, by multivariate analysis of EEG, identify tightly coupled brain modules, which are communicating between themselves by less dense connections. The above approach allows to identify these modules topographically and to estimate quantitatively the ratios of the strength of short- range to long- range connections.

Inspecting results of connectivity analysis by DTF we observe that obtained networks are far from randomness, they may be called deterministic in the sense that they show clear and repeatable structure with well defined sources of activity and distinct (albeit varying in time) patterns of connections. We are not criticizing the approach of graph theoretical analysis in general, because it is a very useful method for analysis of large networks of neurons—real or simulated, also its application to anatomical connections, which form a very dense network, is well justified. We are challenging its application to connectivity patterns obtained from pair-wise connectivity obtained from scalp recorded signals. In a given behavioral condition only some specific connections are active and randomness found in many works is an artifact of the method.

## Conclusions

In our opinion there are two fundamental problems with application of graph theoretical analysis for connectivity estimation. The first one is connected with the fact that application of bivariate methods followed by giving all connections equal weights leads to the dense, disordered, similar to random patterns of connections, from which some “small word” properties may be possibly extracted with some effort. Graph theoretical analysis is used with the aim to find some order in the disorganized structure of connections usually found by bivariate methods. Equal weights are given to all connections (even these very weak ones, without statistical significance), in order to comply with the requirements of graph theoretical analysis assuming that the networks should be fully connected (each node of the network should be connected with each one at least by one path). In the procedure of giving equal weights to all connections the information on the brain connectivity structure is further blurred, namely important information coded in the weights of connections is lost. Sometimes weighted graphs are considered, however in case of connectivity computed by bivariate measures the overall connectivity pattern is still blurred.

The second problem, which is a consequence of the first one, is that graph analysis based on bivariate measures supplies, in fact, very limited information, concerning only the degree to which brain networks are more or less random, more or less “small world”. The attempts to find some topographical or spectral information usually yield the results, which have no correspondence with the evidence coming from other techniques. Therefore we may conjecture that conventional graph analysis based on pair-wise connectivity doesn't give the reliable information on spectral and topographical features of brain connectivity.

The multivariate estimators, which are free from spurious connections, provide very sparse, specific, well determined patterns of brain connectivity. Moreover, these patterns are compatible with the known anatomical, imaging and electrophysiological evidence. More examples of the agreement of the multivariate causal measures of brain connectivity with the other evidence may be found in: [46], [49], [50]. Therefore we may conjecture that neural networks are deterministic in the sense that, for a given task, they reveal specific, well determined patterns of connections.

Multivariate methods offer rich information about the connectivity including its dynamical aspects. They allow to: 1) identify topographical location of centers of increased connectivity, 2) estimate quantitatively intra-modular and inter-modular coupling and 3) find the time-varying patterns of connectivity. Summarizing: we may conjecture that the connectivity structure of brain networks is far from randomness and that multivariate methods and especially methods based on Granger causality should be recommended for connectivity studies.
